# Activation of Inflammatory and Pro-Thrombotic Pathways in Acute Stress Cardiomyopathy

**DOI:** 10.3389/fcvm.2017.00049

**Published:** 2017-08-03

**Authors:** Timothy P. Fitzgibbons, Yvonne J. K. Edwards, Peter Shaw, Aline Iskandar, Mohamed Ahmed, Josiah Bote, Tejen Shah, Sumita Sinha, Robert E. Gerszten, John F. Keaney, Michael R. Zile, Gerard P. Aurigemma

**Affiliations:** ^1^Department of Medicine, University of Massachusetts Medical School, Worcester, MA, United States; ^2^Program in Molecular Medicine, University of Massachusetts Medical School, Worcester, MA, United States; ^3^Division of Cardiovascular Medicine, Maine Medical Center, Portland, ME, United States; ^4^Department of Medicine, Beth Israel Deaconess Medical Center, Boston, MA, United States; ^5^Ralph H. Johnson Veterans Administration Medical Center, Medical University of South Carolina, Charleston, SC, United States

**Keywords:** acute myocardial infarction, coagulation, inflammation, stress cardiomyopathy, women

## Abstract

Stress cardiomyopathy (SCM) is a unique cardiac disorder that more often occurs in women. SCM presents in a similar fashion as acute myocardial infarction (AMI), with chest pain, ECG changes, and congestive heart failure. The primary distinguishing feature is the absence of thrombotic coronary occlusion in SCM. How this reduction in cardiac function occurs in the absence of coronary occlusion remains unknown. Therefore, we tested the hypothesis that a targeted proteomic comparison of patients with acute SCM and AMI might identify relevant mechanistic differences. Blood was drawn in normal controls (*n* = 6), women with AMI (*n* = 12), or women with acute SCM (*n* = 15). Two-week follow-up samples were available in AMI (*n* = 4) and SCM patients (*n* = 11). Relative concentrations of 1,310 serum proteins were measured in each of the 48 samples using the SOMAscan assay. Women with AMI had greater myocyte necrosis, as reflected by a higher peak troponin I concentration (AMI 32.03 ± 29.46 vs. SCM 2.68 ± 2.6 ng/ml, *p* < 0.05). AMI and SCM patients had equivalent reductions in left ventricular ejection fraction [LVEF (%) 39 ± 12 vs. 37 ± 12, *p* = 0.479]. In follow-up, women with SCM had a greater improvement in cardiac function [LVEF (%) 60 ± 7 vs. 45 ± 13, *p* < 0.001]. No differentially expressed proteins were detected (absolute log2-fold change >1; *q* < 0.05) between AMI and SCM in the acute or recovery phase. However, when we compared normal controls to patients with AMI, there was differential expression of 35 proteins. When we compared normal controls to patients with SCM, 45 proteins were differentially expressed. In comparison to normal controls, biological processes such as complement, coagulation, and inflammation were activated in both AMI and SCM. There were four proteins that showed a non-significant trend to be increased in acute SCM vs. AMI (netrin-1, follistatin-like 3, kallikrein 7, kynureninase). Despite a lesser degree of myocardial necrosis than AMI, SCM is characterized by a similar activation of inflammatory, complement, and coagulation pathways. These findings may explain reported thromboembolic complications in the short term and elevated risk of mortality in the long term of SCM.

## Introduction

Stress cardiomyopathy (SCM) is a unique form of heart disease that primarily affects women. Described in 1990 by Sato and colleagues, it is now recognized as accounting for 5.9–7.5% of women presenting to the emergency department with acute coronary syndromes (1–4). Although the prognosis is typically good, complications such as heart failure, arrhythmias, and thromboembolic phenomena do occur (5). Recent studies have shown that SCM patients have increased long-term morbidity and mortality (5.6% risk of death per patient year) (6–8). Therefore, this disorder may not be as benign as previously thought.

After almost 30 years of study, the mechanism of SCM is still not understood. Plasma concentrations of norepinephrine, epinephrine, and dopamine are twofold higher than those of AMI (9). Myocardial biopsy in SCM reveals inflammation and myocyte contraction band necrosis, which are features of catecholamine excess (9). In contrast, acute myocardial infarction (AMI) has greater myocyte necrosis (10). Despite these different mechanisms of injury, cardiac function is impaired to an equivalent degree in both conditions (11). The exact mechanism by which LV dysfunction occurs in SCM remains poorly understood.

Due to the presenting signs and symptoms, patients with SCM are referred for urgent coronary angiography, which reveals the absence of atherosclerotic plaque rupture. Coronary angiography exposes the SCM patient to unnecessary risk without therapeutic benefit. A biomarker that could differentiate between AMI and SCM would be useful, allowing for the identification of low risk SCM patients in whom angiography could be avoided. This would be especially helpful in patients with “secondary” SCM (5). “Secondary SCM” frequently occurs in medical intensive care units and is precipitated by critical illnesses such as sepsis (12). Due to the severity of illness in these cases, cardiac interventions such as coronary angiography or the initiation of antiplatelet therapy are often deferred.

There have been no studies examining novel protein biomarkers in SCM. A ratio of serum B-type natriuretic peptide to troponin I (TnI) ≥1,272 is highly specific for SCM (95%) but has limited sensitivity (52%) (13). Soluble suppression of tumorigenicity 2 has been studied to identify SCM in medical intensive care units (12). A circulating profile of plasma microRNAs (miR-16, mir-26a, miR-1, and miR-133a) has shown good sensitivity and specificity for SCM (14). However, the use of plasma microRNAs for diagnostic purposes has not been adopted in clinical practice (15). Furthermore, these studies shed no light into the biologic differences between these two syndromes.

We hypothesized that there would be differences between circulating proteins in SCM and AMI that might elucidate the underlying mechanisms. Therefore, the purpose of our study was twofold. The first aim was to perform an unbiased comparison of circulating proteins in SCM and AMI in order to provide insight into the pathophysiology of SCM. Our second aim was to identify putative protein biomarkers of SCM.

In order to achieve these aims, we utilized recently described DNA aptamer technology to perform our screen (SOMAscan V1.3K, SOMAlogics) (16–18). This assay uses single stranded DNA aptamers to specifically bind 1,310 predetermined proteins in a single sample. The advantages of this technology include higher throughput and precision than traditional proteomic methods such as mass spectrometry or antibody based methods (16, 17).

Using this assay, we found that women presenting with either SCM or AMI have an increase in proteins related to complement, coagulation, and inflammation. The unexpected and profound activation of inflammatory and pro-thrombotic pathways in SCM may contribute to the increased risk of mortality attributed to this disorder (7, 8).

## Materials and Methods

### Patient Enrollment

Women who presented with non-ST or ST elevation myocardial infarction and had coronary angiography demonstrating either culprit LAD disease or apical variant SCM were prospectively enrolled. Detailed presenting features of the AMI group are shown in Table 1. We specifically limited our study to patients with culprit LAD disease and apical SCM in order to control for the differences between echocardiograms and clinical features that have been demonstrated with variants of SCM (19). All of the SCM patients met the Mayo Clinic Criteria for SCM (20). The normal control group for proteomic analyses consisted of 6 subjects (3 females, 3 males) with a mean age of 51.33 ± 10.11 years. They were healthy ambulatory persons without diabetes, hyperlipidemia, hypertension, or known coronary disease and taking no prescription medications. Written informed consent was obtained according to protocol #00000941 as approved by the University of Massachusetts Medical School IRB.

**Table 1:** Angiographic characteristics of acute myocardial infarction group.

Patient	Culprit lesion	Flow	Presentation	Onset of pain (h)	Peak troponin I (ng/ml)	Treatment
1	100% pLAD	TIMI 0	STEMI	10	90.0	1 DES
2	90% pLAD ulcerated		NSTEMI		1.84	1 DES
3	95% mLAD ulcerated	TIMI 3	STEMI	>48	33.7	2 DES
4	pLAD dissection	TIMI 3	STEMI	4	20.2	None
5	pLAD 40%, possible spasm	TIMI 3	NSTEMI	10	10.8	None
6	pLAD 80%, hazy	TIMI 3	NSTEMI	2	1.21	1 DES
7	pLAD 80%	TIMI 2	NSTEMI		13.8	1 DES
8	D1, 95%	TIMI 2	STEMI	4	35.7	POBA D1
9	pLAD 99%, thrombus	TIMI3	STEMI	>48	70.0	1 DES
10	pLAD 99%, thrombus		STEMI	10	13.0	1 DES
11	pLAD 95%	TIMI 2	STEMI	>48	8.0	1 DES
12	pLAD 95%, hazy		STEMI	1	70.0	1 DES

### Serum Isolation and Proteomic Analysis

Non-fasting venous blood was drawn (after cardiac catheterization but within 24 h of presentation) into SST tubes, spun, and frozen at −80°C. Acute samples were analyzed in 12 AMI patients and 15 SCM patients. Follow-up samples were drawn in clinic at 2 weeks follow-up (AMI *n* = 4, SCM *n* = 11). We chose to sample blood at 2 weeks follow-up because this is the median time to recovery of LV function in SCM (9). Samples were shipped to an outside laboratory on dry ice where assays were performed using SOMAscan reagents according to the manufacturer’s protocol (16). The menu of the 1,310 protein aptamers included in the assay is available online.^1^ The median intra-assay and inter-assay coefficient of variation was 2.1%.

### Echocardiography

Complete 2D echocardiograms were performed within 24 h of presentation and in follow-up (2 weeks) in patients with AMI and SCM. Ejection fraction, 2D volumes, and linear dimensions were measured according to ASE guidelines (21).

### Statistical Methods

Somalogics SOMAScan technology was used to measure levels of 1,310 predetermined proteins included in the assay (see text footnote 1) in 48 patient samples using 1,310. Protein expression levels were expressed as normalized counts [relative fluorescent units (RFUs)] for the 48 patient samples. Data were analyzed using Somalogics’ SomaSuite software (version 1.0.3a). The non-parametric two-sided Mann–Whitney *U*-test was used to analyze the normalized expression levels, to determine statistically significant differentially expressed proteins for each pairwise group comparison. The *p*-value, *q*-value, and other statistics were calculated for each protein. The *q*-values are calculated using the false discovery rate (FDR) method (22). The fold change values for each pairwise comparison are computed on the average values in the log2 scale. Statistically differentially expressed proteins were defined if the following criteria are met (absolute log2-fold change >1; *q* < 0.05). The raw data for this study were deposited in the Gene Expression Omnibus and can be downloaded at https://www.ncbi.nlm.nih.gov/geo/info/linking.html (Accession GSE95368).

In order to determine signaling pathways altered in each condition, functional enrichment analysis was carried out using the WEB-based GEne SeT AnaLysis Toolkit (Webgestalt) (23). The Kyoto Encyclopedia of Genes and Genomes database was selected for enrichment analysis, with the proteome selected as the reference set. The hypergeometric test was used for enrichment evaluation for the lists of statistically differentially expressed proteins. The Benjamini and Hochberg method was used to calculate the adjusted *p*-values (*q*) and the significance cutoff filter was set to *q* < 0.05 (24).

**Figure 1:**
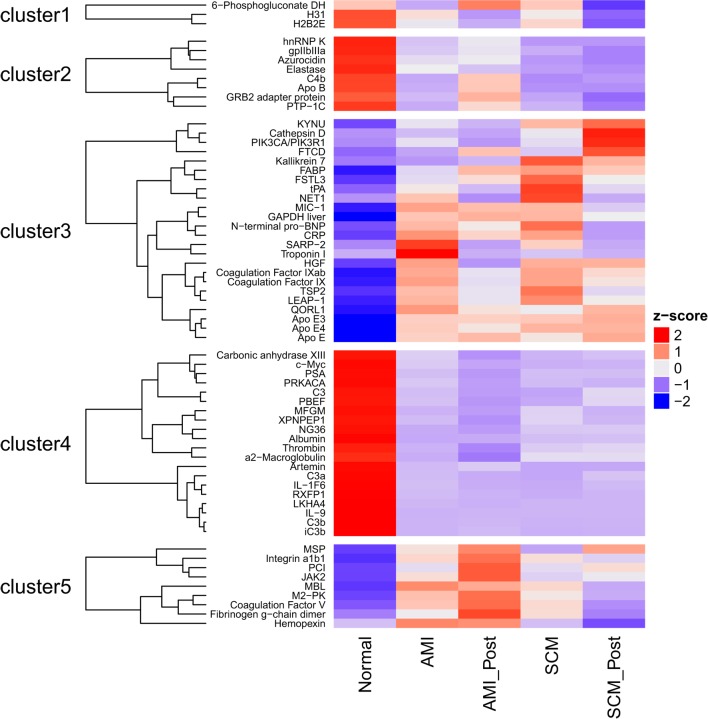
The heatmap shows the serum protein expression in the five groups [normal, the acute myocardial infarction conditions (AMI, AMI_Post) and the stress cardiomyopathy conditions (SCM, SCM_Post)]. By determining if a protein was differentially expressed in one or more of the ten possible comparisons, a list of 64 proteins was generated. Differentially regulated proteins were defined using the following filters (absolute log2 fold change >1; *q* < 0.05). The average level of each protein, in each of these conditions, was used to generate the heat-map. Bright red is increased expression, and deep purple is decreased expression (*Z*-score). For example, concentrations of N-terminal pro-BNP (cluster 3) are increased in AMI and SCM compared to normal.

The complex heatmap package (version 1.12.0) was used to generate a heatmap (Figure 1) and the following parameters were used (clustering distance rows = “maximum,” clustering method rows = “ward.D”) (25). By determining if a protein was differentially expressed in one or more of the 10 possible comparisons (Figure 1), a list of proteins of interest was curated. This list comprising 64 proteins, together with the protein expression levels averaged for each protein in each of the medical conditions, was used as input to generate a heatmap. Five clusters are generated; for each cluster, the members were identified (Figure 1).

## Results

### Clinical Characteristics

The baseline clinical and echocardiographic characteristics are shown in Tables 1–3. Compared to AMI, women with SCM had lower systolic and diastolic blood pressure on admission (112.2 ± 16.5 vs. 126.3 ± 21.9 and 66.4 ± 12.1 vs. 74.8 ± 11.6 mm Hg, *p* < 0.05 for both). Left ventricular end diastolic pressure was elevated in both groups (18.7 ± 9.4 vs. 14.9 ± 14.3 mm Hg, *p* = 0.36). Women with AMI had higher peak values of TnI (32.03 ± 29.46 vs. 2.68 ± 2.6 ng/ml, *p* < 0.05) and CPK (1,206 ± 1,348 vs. 104 ± 63.5 ng/ml, *p* < 0.05) during their hospitalization.

**Table 2:** Clinical characteristics of study participants.

	Acute myocardial infarction (*n* = 16)	Stress cardiomyopathy (*n* = 26)	*p-*Value
Age (years)	57.87 ± 16.0	65.08 ± 9.11	0.12
Height (cm)	158.2 ± 5.8	162.1 ± 6.5	0.06
Weight (kg)	72.3 ± 16.2	72.8 ± 11.6	0.90
BSA (m^2^)	1.7 ± 0.1	1.7 ± 0.14	0.55
Presenting HR (bpm)	80.3 ± 15.9	84.1 ± 14.1	0.43
Presenting SBP (mm Hg)	126.3 ± 21.9	112.2 ± 16.5	*<0.05*
Presenting DBP (mm Hg)	74.8 ± 11.6	66.4 ± 12.1	*<0.05*
HDL (mg/dl)	55.2 ± 36.6	61.3 ± 25.8	0.57
Total cholesterol (mg/dl)	182.4 ± 50.1	172.7 ± 40.8	0.54
LDL (mg/dl)	108.5 ± 44.2	92.5 ± 32.2	0.23
Peak troponin (ng/ml)	32.0 ± 29.4	2.6 ± 2.6	*<0.05*
Peak CPK (ng/ml)	1,206.0 ± 1,348	104.5 ± 63.5	*<0.05*
LVEDP (mm Hg)	14.9 ± 14.3	18.7 ± 9.4	0.36
Emotional stress trigger	0 (0%)	13 (52%)	*<0.001*
Chest pain	14 (93%)	21 (84%)	0.388
Shortness of breath	6 (40%)	11 (44%)	0.804
Syncope	0 (0%)	2 (8%)	0.261
Rales	3 (20%)	0 (0%)	*<0.05*
JVD	2 (13%)	0 (0%)	0.061
S3 or S4	1 (7%)	0 (0%)	0.191
Peripheral edema	4 (27%)	2 (8%)	0.109
Beta blockers	3 (25%)	8 (32%)	0.411
Calcium channel blockers	1 (7%)	5 (20%)	0.253
ACEi/ARBs	3 (25%)	11 (44%)	0.123
Diuretics	3 (25%)	6 (24%)	0.769
Statins	1 (7%)	11 (44%)	*<0.05*
ASA	4 (27%)	9 (36%)	0.542
SSRI	3 (18%)	9 (34%)	0.282
Hypertension	6 (40%)	17 (68%)	0.083
Diabetes mellitus	3 (25%)	5 (20%)	0.281
Smoking history	8 (53%)	8 (32%)	0.182
Depression/anxiety	6 (40%)	14 (56%)	0.327

*BSA, body surface area; HR, heart rate; SBP, systolic blood pressure; DBP, diastolic blood pressure; HDL, high-density lipoprotein; LDL, low-density lipoprotein; CPK, creatinine phosphokinase; LVEDP, left ventricular end-diastolic pressure; JVD, jugular venous distension; S3 or S4, third or fourth heart sound, respectively; ACEi/ARB, angiotensin converting enzyme inhibitor/angiotensin receptor blocker; ASA, aspirin; SSRI, selective serotonin reuptake inhibitor*.

*Values are mean ± SD or *n* (%). Italicized p values are statistically significant*.

**Table 3:** Acute and recovery echocardiographic data.

	Acute myocardial infarction (*n* = 11)	Stress cardiomyopathy (*n* = 15)	*p*-Value

	Mean ± SD	Mean ± SD	
**Acute**			
EF (%)	39 ± 12	37 ± 12	0.479
LAVI (ml/m^2^)	33.1 ± 7.1	27.25 ± 6.0	*<0.05*
LVMI (gm/m^2^)	81.55 ± 19.0	82.95 ± 21.0	0.53
RWT	0.38 ± 0.04	0.41 ± 0.06	0.51
LVIDd (mm)	47.45 ± 5.99	45.58 ± 6.54	0.234
LVIDs (mm)	34.11 ± 7.27	30.05 ± 7.06	*<0.05*
LVEDV (ml)	95.15 ± 25.67	77.11 ± 23.70	*<0.001*
LVESV (ml)	56.73 ± 25.19	43.17 ± 12.94	*<0.01*
LVEDVI (ml/m^2^)	54.50 ± 13.63	45.53 ± 13.60	*<0.001*
LVESVI (ml/m^2^)	32.34 ± 13.54	24.41 ± 7.56	*<0.001*
**Recovery**			
EF (%)	45 ± 13	60 ± 7	*<0.001*
ΔEF (%)	+8 ± 13	+23 ± 12	*<0.001*
LVIDd (mm)	49.91 ± 7.73	45.50 ± 5.58	*<0.05*
ΔLVIDd (mm)	+1.40 ± 8.11	−0.21 ± 6.61	0.450
LVIDs (mm)	35.18 ± 8.30	27.68 ± 4.82	*<0.001*
ΔLVIDs (mm)	−1.37 ± 7.46	−2.54 ± 5.17	0.529
LVEDV (ml)	99.88 ± 36.93	75.50 ± 18.93	*<0.001*
ΔLVEDV (ml)	+5.77 ± 26.9	−3.20 ± 25.40	0.320
LVESV (ml)	58.82 ± 34.09	34.81 ± 13.67	*<0.001*
ΔLVESV (ml)	+1.25 ± 13.34	−10.60 ± 15.70	*<0.05*
LVEDVI (ml/m^2^)	57.38 ± 18.57	45.50 ± 11.28	*<0.001*
ΔLVEDVI (ml/m^2^)	+1.71 ± 15.38	−0.32 ± 14.03	0.917
LVESVI (ml/m^2^)	33.43 ± 17.87	19.63 ± 7.17	*<0.001*
ΔLVESVI (ml/m^2^)	+2.03 ± 13.19	−4.95 ± 9.7	*<0.05*

*EF, ejection fraction; LAVI, left atrial volume index; LVMI, left ventricular mass index; RWT, relative wall thickness; LVIDd, left ventricular internal dimension diastolic; LVIDs, left ventricular internal dimension systolic; LVEDV, left ventricular end diastolic volume; LVESV, left ventricular end systolic volume; LVEDVI, left ventricular end diastolic volume index; LVESVI, left ventricular end systolic volume index; ΔEF (%), change in ejection fraction; ΔLVIDd, change in left ventricular internal dimension diastolic; ΔLVIDs, change in left ventricular internal dimension systolic; ΔLVEDV, change in left ventricular end diastolic volume; ΔLVESV, change in left ventricular end systolic volume; ΔLVEDVI, change in left ventricular end diastolic volume index; ΔLVESVI, change in left ventricular end systolic volume index*.

*Values are mean ± SD or *n* (%). Italicized p values are statistically significant*.

### Echocardiographic Characteristics

Stress cardiomyopathy and AMI patients had equivalent reductions in systolic function [LVEF (%)] in the acute phase (37 ± 12 vs. 39 ± 12, *p* = 0.47) (Table 3). However, SCM patients had smaller indexed left ventricular systolic (LVESVI 24.41 ± 7.56 vs. 32.34 ± 13.54 ml/m^2^, *p* < 0.001) and diastolic (LVEDVI 45.53 ± 13.60 vs. 54.50 ± 13.63 ml/m^2^, *p* < 0.001) volumes than AMI. SCM patients also had a greater indexed left atrial volume than AMI (LAVI 33.1 ± 7.7 vs. 27.25 ± 6.0, *p* < 0.05).

Stress cardiomyopathy patients had a significantly greater improvement in LV systolic function in follow-up, as shown by the mean LVEF (%) (60 ± 7 vs. 45 ± 13, *p* < 0.001), interval change in ΔLVEF (%) (+23 ± 12 vs. +8 ± 13, *p* < 0.001), and interval reduction in LVESVI (ΔLVESVI −4.95 ± 9.7 vs. +2.0 ± 13.19 ml/m^2^, *p* < 0.05).

### Proteomic Analyses

There were no proteins differentially expressed (absolute log2-fold change >1; *q* < 0.05) between AMI and SCM in the acute phase (Table 4). This was true even when we lowered the threshold for differential regulation to *q* < 0.05 with any fold change (Table 4). When we compared normal controls vs. AMI, there were 35 proteins with differential expression (absolute log2-fold change >1; *q* < 0.05) (Figure 1; Table 4). In the normal vs. SCM comparison, 45 proteins were differentially regulated (absolute log2-fold change >1; *q* < 0.05) (Figure 1; Table 4).

**Table 4:** Number of differentially expressed (DE) proteins between pairwise comparisons.

Conditions compared	Number of DE proteins with *q* < 0.05 (absolute log2-fold change ignored)	Number of DE proteins with absolute log2-fold change >1; *q* < 0.05	Number of DE proteins with absolute log2-fold change >1; *q* < 0.05	Number of DE proteins with absolute log2-fold change >1; *q* < 0.05
			Downregulated in condition 1	Upregulated in condition 1
Normal vs. acute myocardial infarction (AMI)	98	35	17	18
AMI vs. AMI post	0	0	0	0
Normal vs. AMI post	0	0	0	0
Normal vs. stress cardiomyopathy (SCM)	124	45	22	23
SCM vs. SCM post	0	0	0	0
Normal vs. SCM post	159	46	17	29
AMI vs. SCM	0	0	0	0
AMI vs. SCM post	40	4	1	3
SCM vs. AMI post	0	0	0	0
AMI post vs. SCM post	0	0	0	0

**Figure 2:**
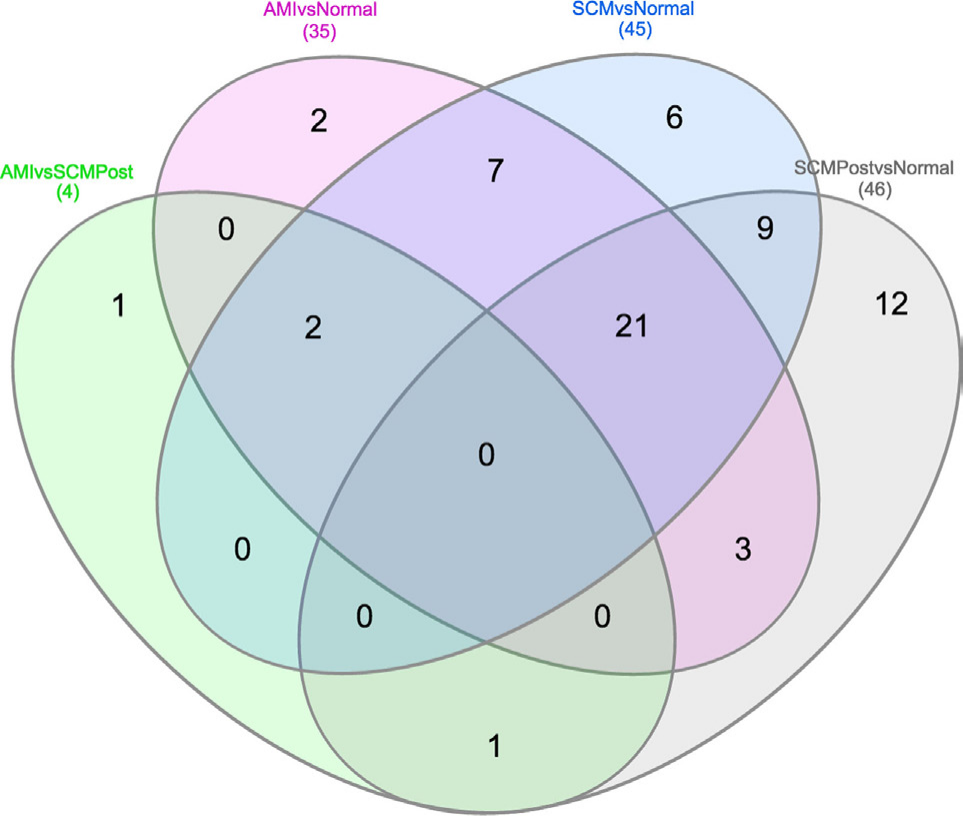
Venn diagram showing differential and overlapping protein expression between the conditions compared (Table 4). As shown in the middle of the diagram, 30 (7**+**21**+**2) proteins are commonly differentially regulated in AMI and SCM compared to normal controls. Specific proteins from Venn diagram subsections are listed in Table 5.

Most of the proteins were commonly differentially regulated in both SCM and AMI compared to controls [Figure 1 (cluster 4), Figure 2; Table 5]. However, there were four candidate proteins that tended to be increased in acute SCM compared to AMI and controls. These proteins were kynureninase (KYNU), K7, follistatin-like 3, and netrin-1 (NET1) [Figure 1 (cluster 3); Table 5].

**Table 5:** Specific proteins from Venn diagram subsection.

Venn diagram segment	Proteins
2 proteins exclusively in acute myocardial infarction (AMI) vs. normal	HAMP, FGG
6 proteins exclusively in stress cardiomyopathy (SCM) vs. normal	C3, F5, FSTL3, Netrin-1, PLAT, THBS2
12 proteins exclusively in SCM post vs. normal	PGD, MYC, CTSD, GRB2, HIST2H2BE, HIST1H3A, IL9, JAK2, Kynureninase, MST1, SERPINA5, PIK3CA/PIK3R1
1 protein exclusively in AMI vs. SCM post	HPX
7 common proteins in AMI vs. normal and SCM vs. normal	NAMPT, F9, F9ab, HGF, GDF-15, ITGA1/ITGB1, MBL2
1 common protein in SCM post vs. normal and AMI vs. SCM post	KLK7
3 common proteins in AMI vs. normal and SCM post vs. normal	XPNPEP1, PTPN6, CRYZL1
21 common elements in AMI vs. normal, SCM vs. normal and SCM post vs. normal	C3, LTA4H, C3a, iC3b, APOB, IL36A, C4A/C4B, ALB, EHMT2, MFGE8, F2, A2M, PRKACA, KLK3, RXFP1, APOE4, APOE3, PKM2, APOE, GAPDH, NPPB
9 common elements in SCM vs. normal and SCM post vs. normal	ARTN, AZU1, CA13, ELANE, FABP3, FTCD, ITGA2B/ITGB3, HNRNPK, SFRP1
2 common elements in AMI vs. normal, SCM vs. normal, and AMI vs. SCM post	CRP, TNNI3

**Figure 3:**
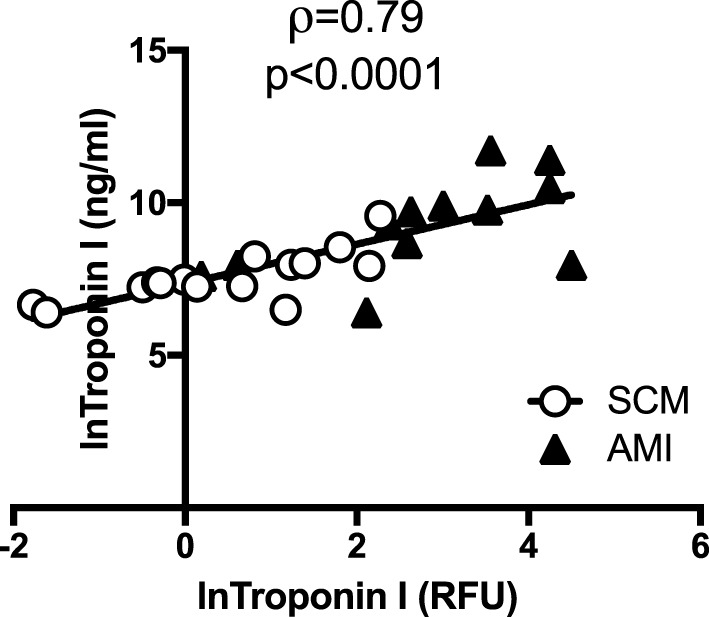
SOMAscan measured troponin correlates well with hospital-based measurement. There was a strong and significant correlation between peak troponin I (TnI) (ng/ml) measured in the hospital laboratory and the TnI [relative fluorescent units (RFU)] as measured by SOMAscan. Open circles represent peak TnI in stress cardiomyopathy (SCM) patients (*n* = 15) and black triangles represent peak TnI in acute myocardial infarction (AMI) patients (*n* = 12).

The heat map (Figure 1) and bar plots of significantly altered proteins (Figures S1 and S2 in Supplementary Material) demonstrated the expected rise and fall of traditional markers of necrosis (TNNI3, CK-MB), inflammation (CRP), and wall stress (NPPB) in the acute and follow-up phase. There was a strong positive correlation between the hospital laboratory measured peak TnI (nanogram per milliliter) and the SOMAscan measured TnI (RFU) (ρ = 0.79, *p* < 0.001) (Figure 3).

Pathway analysis of the differentially regulated proteins in each group revealed a large degree of similarity (Tables 6 and 7). The complement and coagulation pathway displayed the greatest enrichment ratio in both groups (Tables 6 and 7). There was significant overlap among other inflammatory pathways, nine of which were commonly increased in SCM and AMI. Several pathways were unique to SCM: focal adhesion, extracellular matrix–receptor interaction, malaria, regulation of actin cytoskeleton, arrythmogenic right ventricular cardiomyopathy, axon guidance, and Wnt signaling (Table 7).

**Table 6:** KEGG pathway enrichment analysis: normal vs. acute myocardial infarction.

KEGG pathway	Proteins	Enrichment ratio	Adj. *p*-value
Complement and coagulation cascades	C4B, C3, FGG, F2, F9, A2M	150.87	<0.001
*Staphylococcus aureus* infection	MBL2, C4B, C3, FGG	108.16	<0.001
Leshmaniasis	C3, PTPN6, ITGB1	61.96	<0.001
Dilated Cardiomyopathy	TNNI3, PRKACA, ITGB1	49.57	<0.001
Phagosome	MBL2, C3, ITGB1	29.16	<0.001
Pathways in cancer	HGF, KLK3, ITGB1	13.69	<0.05
Hypertrophic cardiomyopathy	TNNI3, ITGB1	35.83	<0.05
Systemic lupus erythematosus	C3, C4B	21.87	<0.05
Alzheimer’s disease	APOE, GAPDH	17.81	<0.05

*KEGG, Kyoto Encyclopedia of Genes and Genomes*.

**Table 7:** KEGG pathway enrichment analysis: normal vs. stress cardiomyopathy.

KEGG pathway	Proteins	Enrichment ratio	Adj. *p*-value
Complement and coagulation cascades	C3, C4B, F2, A2M, F9, PLAT, F5	131.59	<0.001
Phagosome	MLB2, C3, THBS2, ITGB3, ITGB1	37.09	<0.001
Dilated cardiomyopathy	TNNI3, PRKACA, ITGB3, ITGB1	50.44	<0.001
*Staphylococcus aureus* infection	C3, C4B	61.90	<0.001
Focal adhesion	THBS2, HGF, ITGB3, ITGB1	22.70	<0.001
Hypertrophic cardiomyopathy	TNNI3, ITGB3, ITGB1	41.02	<0.001
ECM–receptor interaction	THBS2, ITGB3, ITGB1	40.06	<0.001
Systemic lupus erythematosus	C3, C4B, ELANE	25.04	<0.01
Malaria	HGF, THBS2	44.51	<0.01
Regulation of actin cytoskeleton	F2, ITGB3, ITGB1	15.98	<0.01
Arrhythmogenic right ventricular cardiomyopathy	ITGB3, ITGB1	30.67	<0.01
Leishmaniasis	C3, ITGB1	31.53	<0.01
Pathways in cancer	HGF, KLK3, ITGB1	10.44	<0.01
Axon guidance	Netrin-1, ITGB1	17.60	<0.05
Wnt signaling pathway	SFRP1, PRKACA	15.30	<0.05
Alzheimer’s disease	APOE, GAPDH	13.59	<0.05

*KEGG, Kyoto Encyclopedia of Genes and Genomes*.

## Discussion

Although the pathophysiology, natural history, and outcomes of AMI and SCM are different, they do share common features. First, both are characterized by acutely decreased cardiac function (11). Second, each condition can be complicated by heart failure, arrhythmia, or thromboembolic events. Due to these similarities, women presenting with SCM are often referred for coronary angiography (5). However, in low risk cases, and in cases of “secondary SCM,” a biomarker would be useful. One of the aims of our study was to discover putative biomarkers of SCM; we identified four candidates (FSTL3, Kallikrein7, KYNU, and NET1). A second aim was to learn about the pathophysiology of SCM by comparing serum proteins to normal patients and those with AMI. We were surprised to find no differences between SCM and AMI in the acute phase. Both conditions were characterized by a robust increase in proteins related to inflammation, coagulation, and complement.

The characteristics of both study groups were consistent with prior studies. Patients with AMI had a greater peak TnI and a lower LVEF (%) on follow-up (7, 9, 11). SCM patients had smaller indexed LV diastolic and systolic volumes in both the acute and recovery phase. SCM patients had normalization of their systolic function in recovery, as demonstrated by a greater increase in LVEF (%) (Table 2).

In regards to our proteomic analysis, we wish to emphasize that the SOMAscan 1.3 K measures a predetermined panel of 1,310 proteins, which is only 6.5% of the approximately 20,000 protein encoding genes in the genome. Therefore, there are many changes that were undetected by our assay. For example, tissue inhibitor of metalloproteinase 4 has previously been shown to be increased in SCM compared to AMI (26). However, tissue inhibitor of matrix metalloproteinase (TIMP)-4 specific aptamers were not included in the version of the SOMAscan that we used; therefore, this difference was not detected. Of the proteins that were included, many were commonly elevated in both AMI and SCM, particularly those related to complement, coagulation, and innate immunity. This was unexpected, as the degree of myocardial necrosis in SCM is less than in AMI (7, 9). The trigger for this acute inflammatory response remains unclear.

Complement is an arm of the innate immune system that aids in the detection and clearance of self and bacterial antigens (27). It was demonstrated in 1990 that complement is activated in AMI (27). This is the first report of complement activation in SCM. Local hypoxia may play a role in complement activation and neutrophil chemoattraction to sites of ischemia (27). Complement activation can trigger endothelial adhesion molecule expression, and subsequent leukocyte rolling, arrest, and diapedesis. It is notable that there are several small studies indicating that patients with SCM have greater endothelial dysfunction than those with AMI even in the subacute phase (28–30). This dysfunction could be due to low-level complement activation, increased leukocyte adhesion, and release of reactive oxygen species.

Complement is known to interact with the coagulation cascade (27). The increased levels of coagulation factor V (F5) and tissue plasminogen activator (PLAT, tPA) in SCM indicate that a pro-thrombotic state may be present. Patients with SCM have inducible hyperviscosity and there are multiple reports of increased thromboembolic events in the acute phase of SCM (31–33).

Several candidate proteins demonstrated a non-significant trend to be increased in acute SCM compared to AMI and controls [NET1, FSTL3, kallikrein 7 (KLK7), KYNU] (Figure 1, cluster 3). Netrin-1 is a neuronal growth factor that was originally reported in 1991 (34). NET1 promotes endothelial cell nitric oxide production and cell growth and migration *in vitro via* the receptor deleted in colorectal cancer (DCC) (35). NET1 has also been shown to reduce infarct size in mice (36). There is interest in using NET1 for the treatment of postischemic conditioning in AMI.

Follistatin-like 3 (FSTL3) was increased in SCM compared to controls. FSTL3 inhibits transforming growth factor beta (TGFβ) signaling (37). FSTL3 is upregulated in the myocardium and endothelium of failing human hearts (38). Cardiac specific ablation of FSTL3 reduces cardiac hypertrophy and fibrosis in response to transverse aortic constriction (37). FSTL3 may contribute to myocyte hypertrophy in response to stress stimuli, and the increased levels of FSTL3 in SCM may promote cardiac hypertrophy and fibrosis. This is consistent with prior data demonstrating that SCM has a matrix metalloproteinase (MMP)/tissue inhibitor of matrix metalloproteinase (TIMP) profile similar to hypertensive heart disease, which is distinct from AMI (26). We observed the same MMP/TIMP profile in our study (data not shown); however, the differences did not reach statistical significance. This is likely because the prior study used ELISA to measure MMP/TIMPs (26).

Kynureninase is an enzyme involved in tryptophan metabolism. It degrades 3-hydroxy kynurenine to 3-hydroxyanthranillic acid (39). Kynurenine is an endothelium-derived relaxation factor induced in sepsis (40). Whether or not KYNU is truly elevated in SCM will be the topic of future studies. Finally, KLK7 is a serine proteinase belonging to the human kallikrein gene family, of which there are 15 members (41). The kallikrein system is broadly expressed and has also been demonstrated to play a role in cardiac remodeling (42).

### Limitations

We acknowledge that our study has a small sample size and this may lead to false negative results. The AMI group included patients with both infarction and ischemia; this led to a large SD in the peak TnI value for the AMI group (Table 1). Differences in medications given during the hospital stay may have influenced our results, in particular, the administration of heparin (17). The majority of our study participants were Caucasian women; therefore, the applicability of other races is limited. Many of our patients are referred from outside hospitals and were not available for the follow-up blood draw and echocardiogram. Unfortunately, we could not run the SOMAscan on all our samples due to cost limitations. Despite these limitations, we feel that our observations are original and make an important contribution to the literature.

## Conclusion

In summary, despite different pathophysiologic mechanisms, the circulating proteome of AMI and SCM is similar. Activation of complement and coagulation pathways in SCM has not been previously reported and highlights the pro-thrombotic and inflammatory state of acute SCM. The stimulus for inflammation in SCM is unclear and will be the subject of future investigation. NET1, FSTL3, and KYNU, and KLK7 are putative markers of SCM that require prospective validation.

## Ethics Statement

This study was carried out in accordance with the recommendations of “Committee for the Protection of Human Subjects in Research” with written informed consent from all subjects. All subjects gave written informed consent in accordance with the Declaration of Helsinki. The protocol was approved by the Institutional Review Board of UMASS Medical Center, IRB docket #00000941.

## Author Contributions

TF designed the study, interpreted the data, drafted and revised the manuscript, approved the final version, and is accountable for all aspects of the work. YE, PS, AI, RG, SS, JB, TS, and MA acquired the data, revised the manuscript, approved the final version, and are accountable for all aspects. JK, MZ, and GA made substantial contributions to the study design, revised the manuscript, approved the final version, and are accountable for all aspects of the work.

## Conflict of Interest Statement

The authors declare that the research was conducted in the absence of any commercial or financial relationships that could be construed as a potential conflict of interest.

## Footnotes

^1^http://www.somalogic.com/Technology/SOMAscan-basic-info/.
