# Supplementary material for: Activation of Inflammatory and Pro-Thrombotic Pathways in Acute Stress Cardiomyopathy
Source: Front Cardiovasc Med. 2017 Aug 3;4:49. doi: 10.3389/fcvm.2017.00049 (PMC5541033; doi:10.3389/fcvm.2017.00049)
Supplement: Supplementary file 1 [file data_sheet_1.pdf]

Supplementary Figure 1. Bar plots for the normal vs. SCM and SCM post comparison. The proteins shown in this figure are the 45 proteins that were significantly different (log2 FC>1; q<0.05) in the normal vs. SCM comparison. Normal (n=6), SCM (n=15), SCM Post (n=11).

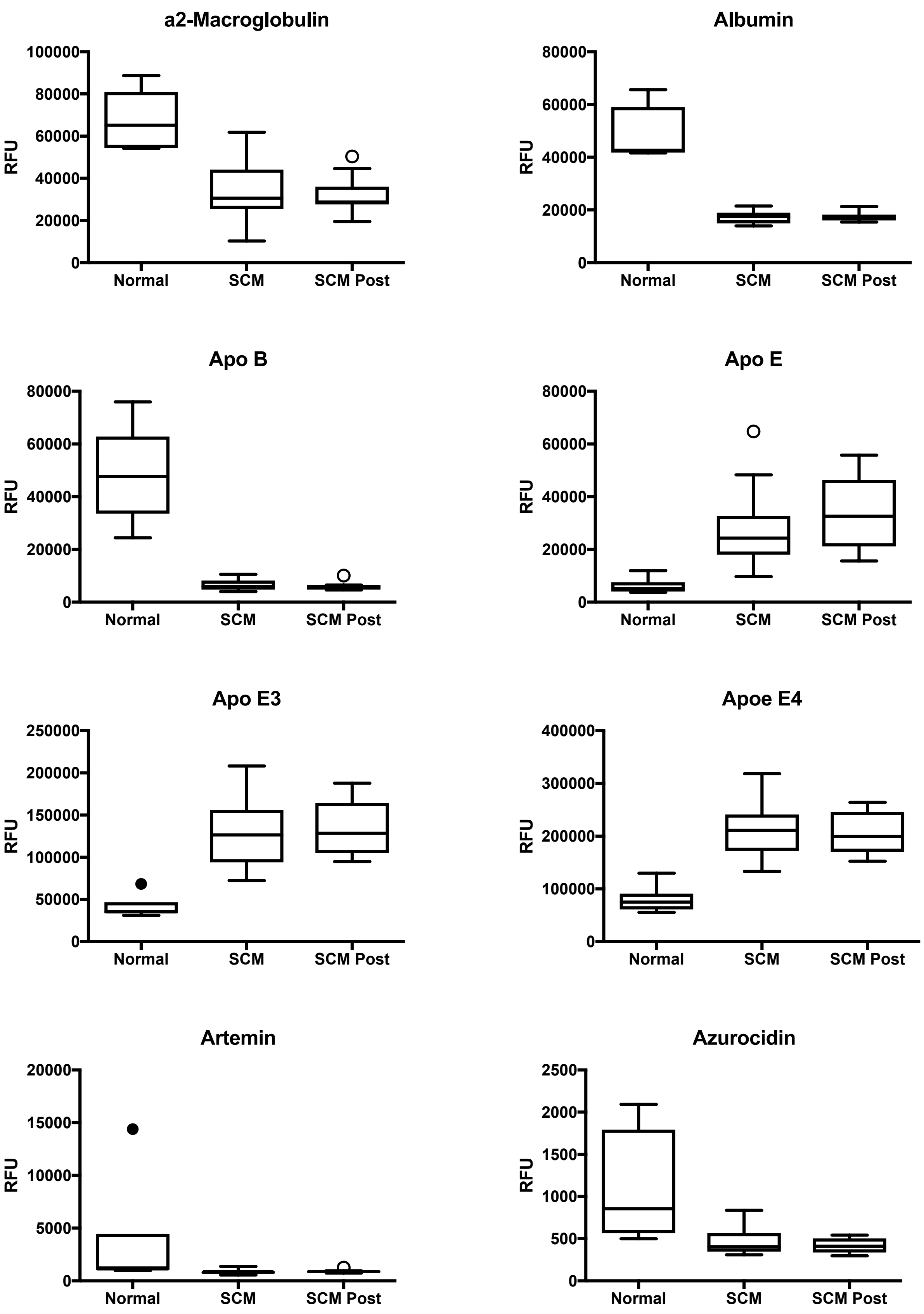
a2-Macroglobulin
RFU
0
20000
40000
60000
80000
100000
Normal
SCM
SCM Post
Albumin
RFU
0
20000
40000
60000
80000
Normal
SCM
SCM Post
Apo B
RFU
0
20000
40000
60000
80000
Normal
SCM
SCM Post
Apo E
RFU
0
20000
40000
60000
80000
Normal
SCM
SCM Post
Apo E3
RFU
0
50000
100000
150000
200000
250000
Normal
SCM
SCM Post
Apoe E4
RFU
0
100000
200000
300000
400000
Normal
SCM
SCM Post
Artemin
RFU
0
5000
10000
15000
20000
Normal
SCM
SCM Post
Azurocidin
RFU
0
500
1000
1500
2000
2500
Normal
SCM
SCM Post

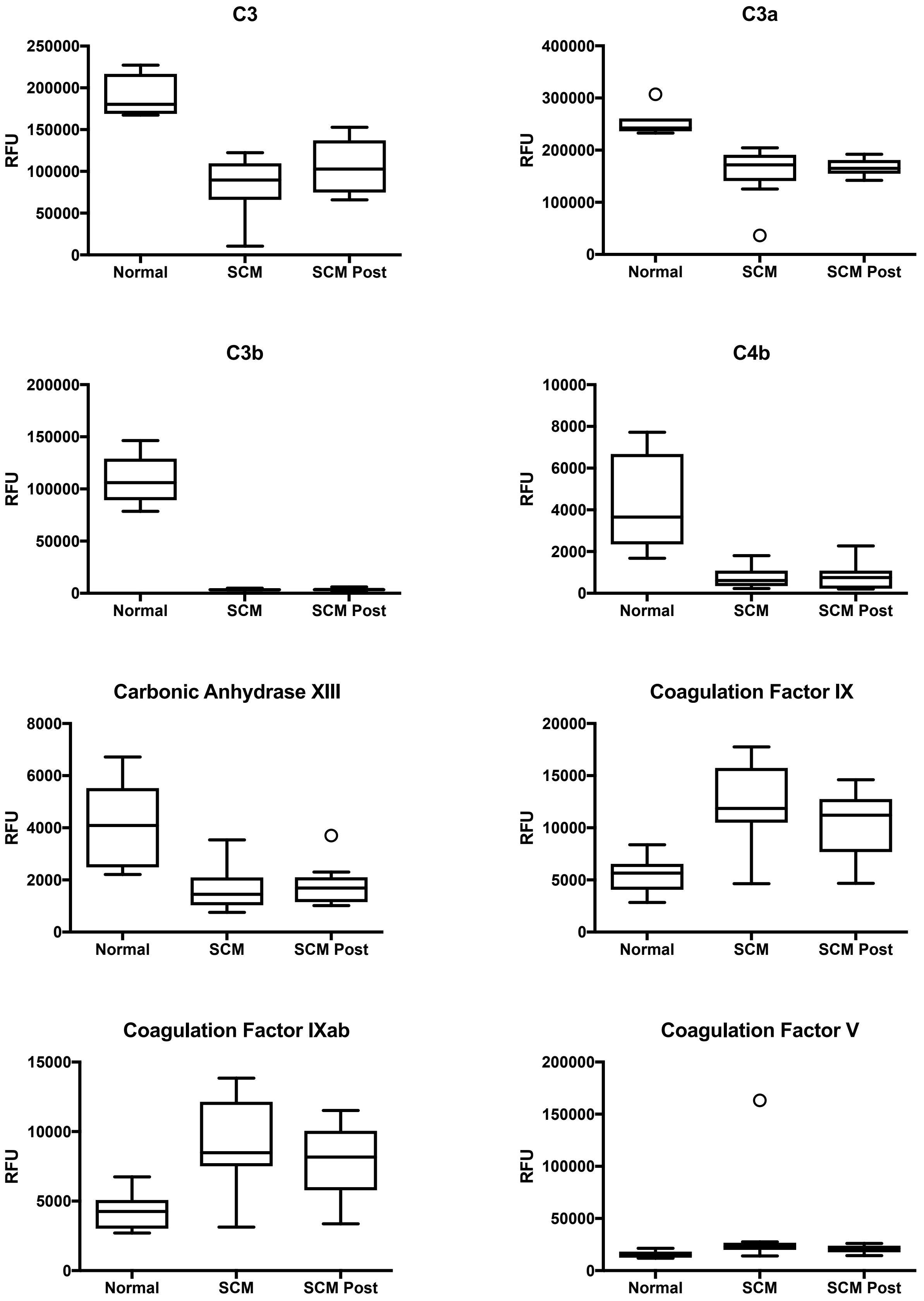

C3
RFU
250000
200000
150000
100000
50000
0
Normal
SCM
SCM Post
C3a
RFU
400000
300000
200000
100000
0
Normal
SCM
SCM Post
C3b
RFU
200000
150000
100000
50000
0
Normal
SCM
SCM Post
C4b
RFU
10000
8000
6000
4000
2000
0
Normal
SCM
SCM Post
Carbonic Anhydrase XIII
RFU
8000
6000
4000
2000
0
Normal
SCM
SCM Post
Coagulation Factor IX
RFU
20000
15000
10000
5000
0
Normal
SCM
SCM Post
Coagulation Factor IXab
RFU
15000
10000
5000
0
Normal
SCM
SCM Post
Coagulation Factor V
RFU
200000
150000
100000
50000
0
Normal
SCM
SCM Post

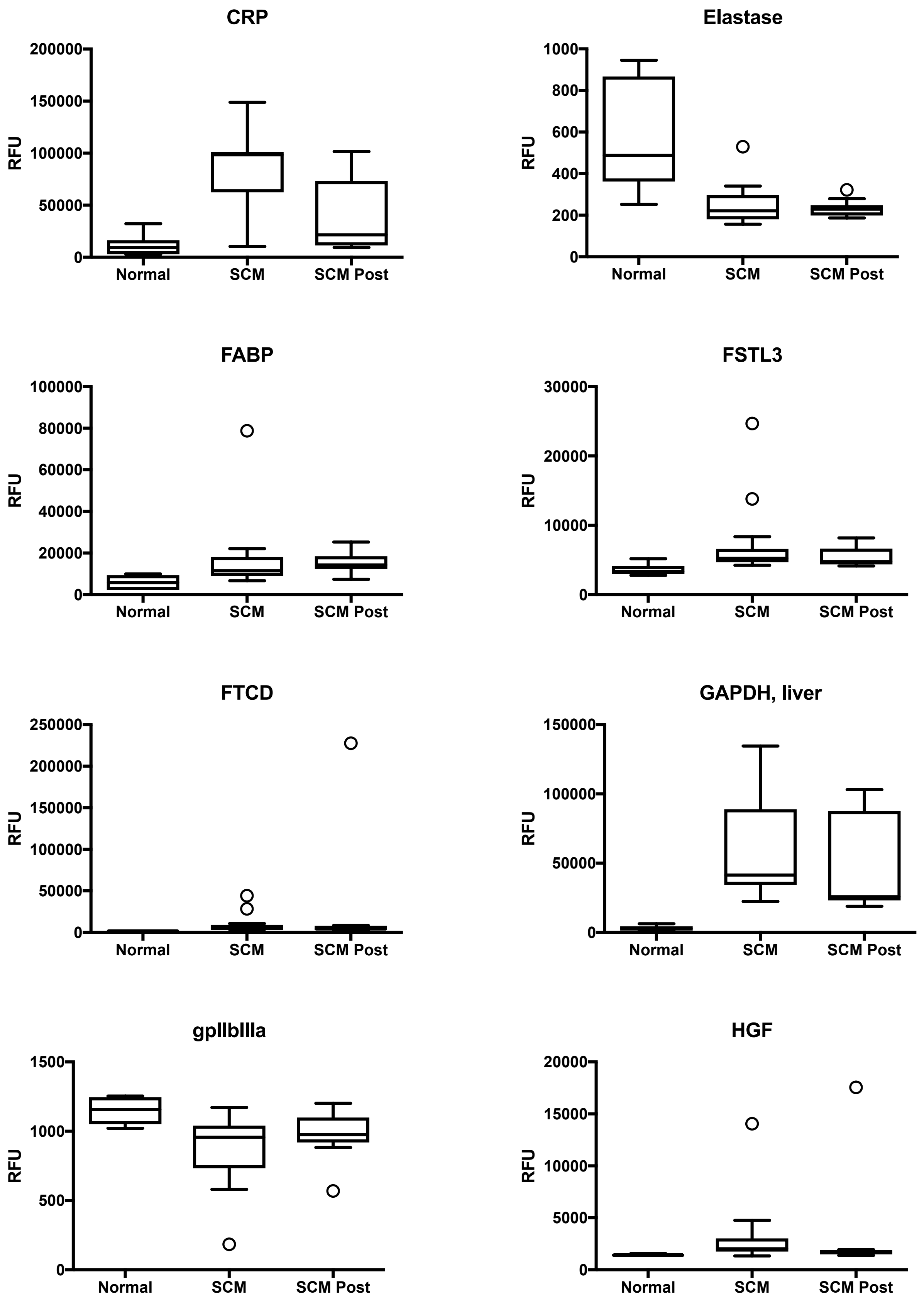

CRP
RFU
200000
150000
100000
50000
0
Normal
SCM
SCM Post
Elastase
RFU
1000
800
600
400
200
0
Normal
SCM
SCM Post
FABP
RFU
100000
80000
60000
40000
20000
0
Normal
SCM
SCM Post
FSTL3
RFU
30000
20000
10000
0
Normal
SCM
SCM Post
FTCD
RFU
250000
200000
150000
100000
50000
0
Normal
SCM
SCM Post
GAPDH, liver
RFU
150000
100000
50000
0
Normal
SCM
SCM Post
gpIIbIIIa
RFU
1500
1000
500
0
Normal
SCM
SCM Post
HGF
RFU
20000
15000
10000
5000
0
Normal
SCM
SCM Post

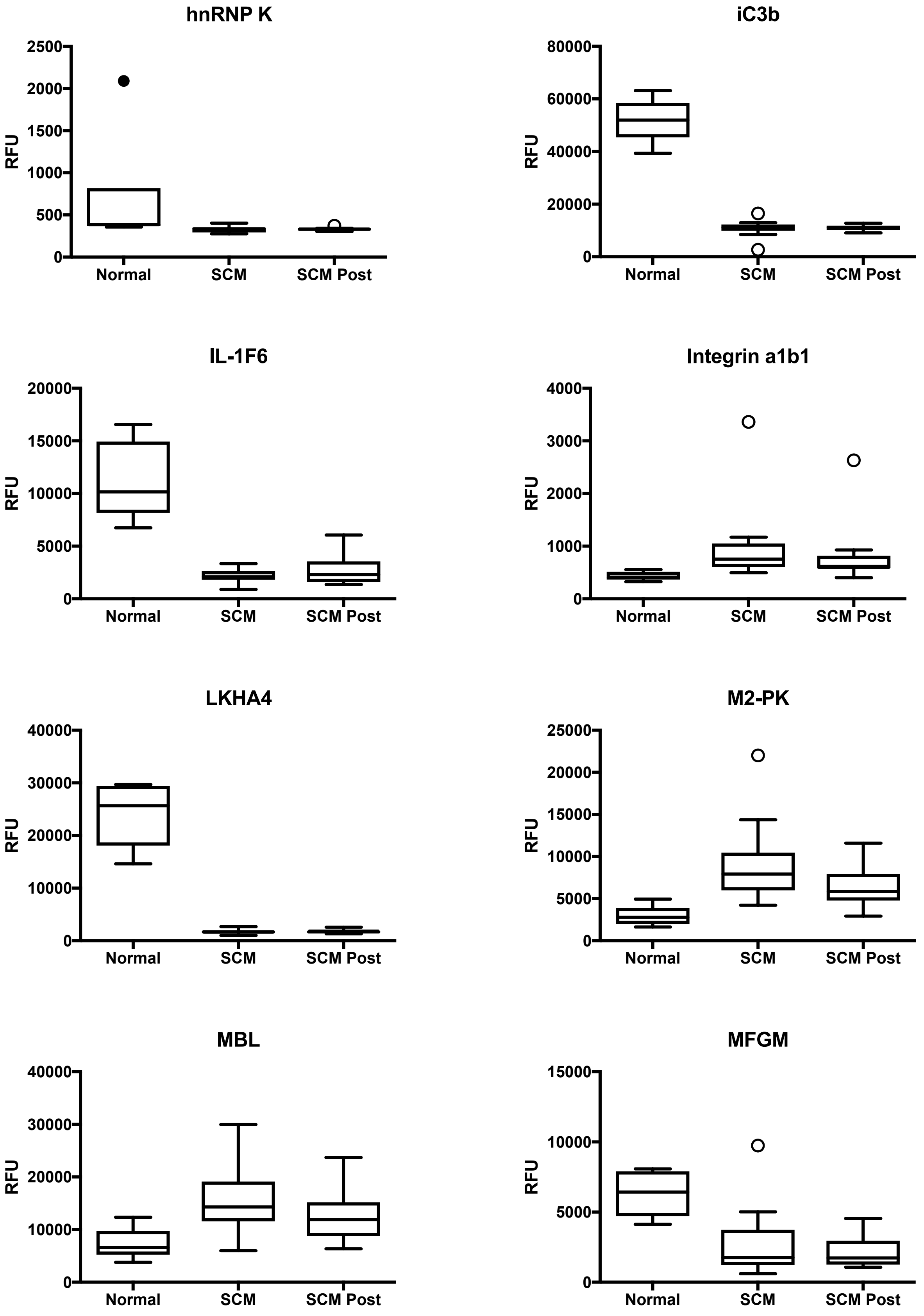
hnRNP K
RFU
2500
2000
1500
1000
500
0
Normal
SCM
SCM Post
iC3b
80000
60000
40000
20000
IL-1F6
20000
15000
10000
5000
Integrin a1b1
4000
3000
2000
1000
LKHA4
40000
30000
M2-PK
25000
MBL
MFGM

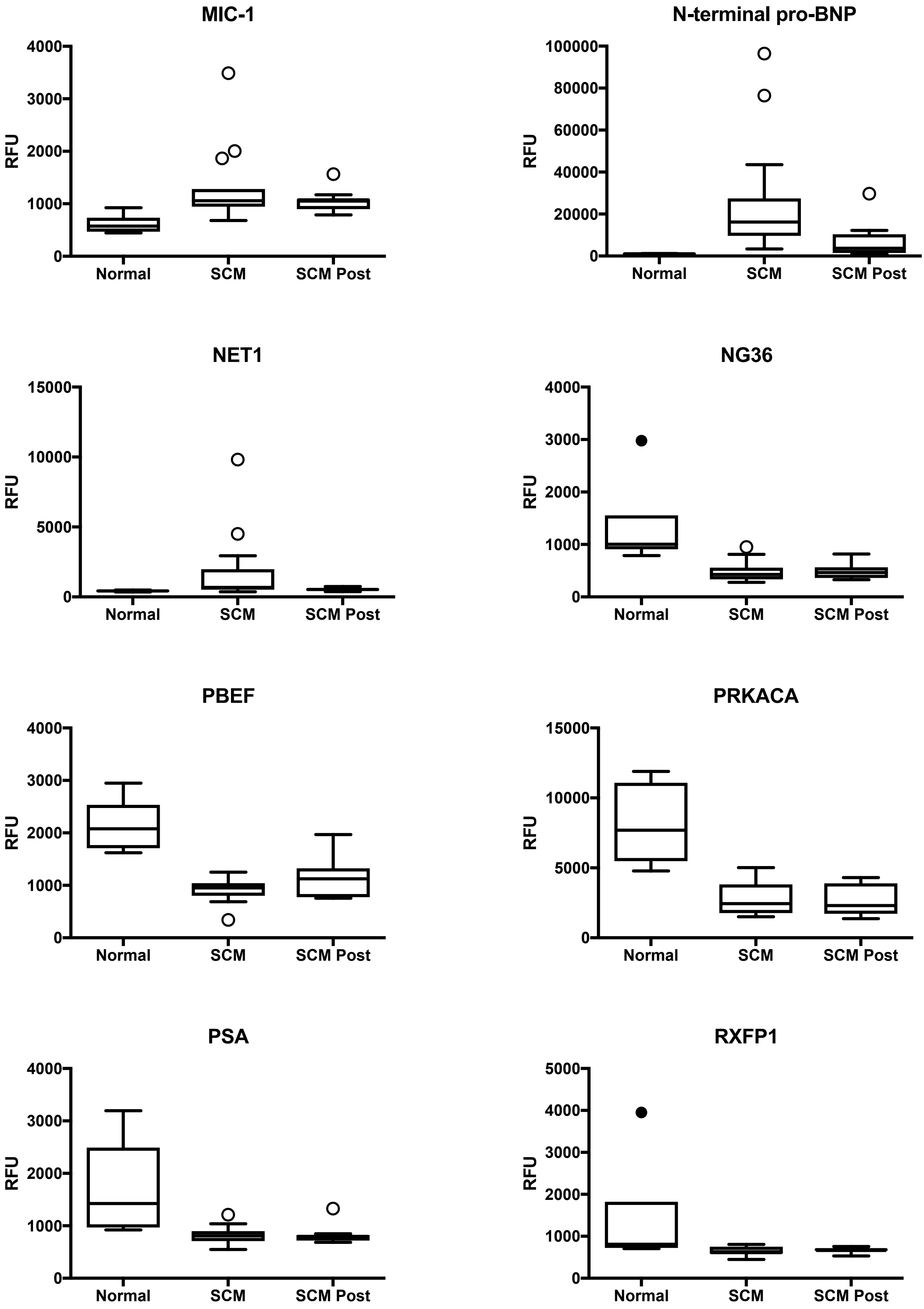
MIC-1
N-terminal pro-BNP
NET1
NG36
PBEF
PRKACA
PSA
RXFP1
RFU
Normal
SCM
SCM Post
0
1000
2000
3000
4000
5000
10000
15000
20000
40000
60000
80000
100000

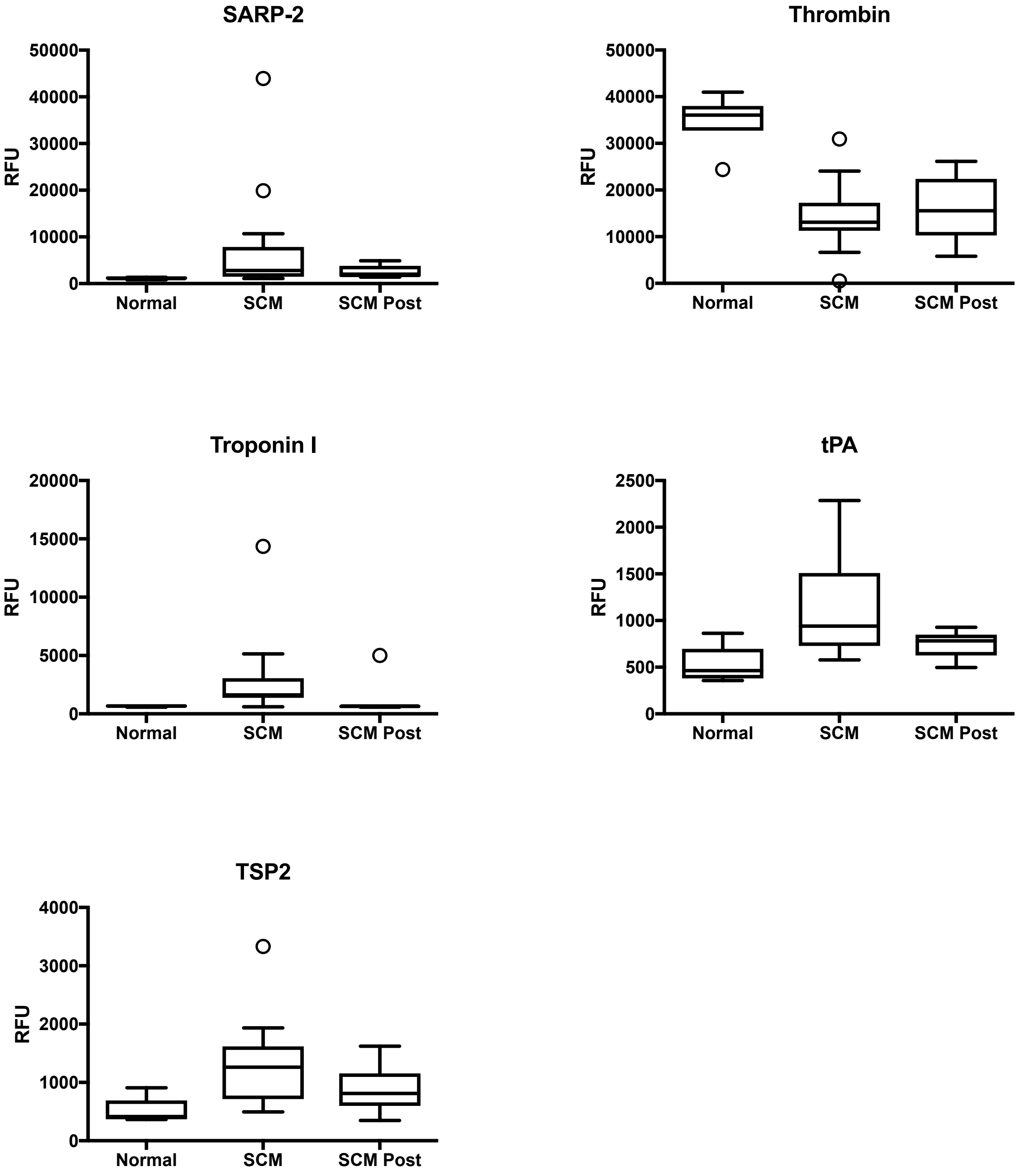
SARP-2
RFU
50000
40000
30000
20000
10000
0
Normal
SCM
SCM Post
Thrombin
RFU
50000
40000
30000
20000
10000
0
Normal
SCM
SCM Post
Troponin I
RFU
20000
15000
10000
5000
0
Normal
SCM
SCM Post
tPA
RFU
2500
2000
1500
1000
500
0
Normal
SCM
SCM Post
TSP2
RFU
4000
3000
2000
1000
0
Normal
SCM
SCM Post
