# Supplementary material for: Activation of Inflammatory and Pro-Thrombotic Pathways in Acute Stress Cardiomyopathy
Source: Front Cardiovasc Med. 2017 Aug 3;4:49. doi: 10.3389/fcvm.2017.00049 (PMC5541033; doi:10.3389/fcvm.2017.00049)
Supplement: Supplementary file 2 [file data_sheet_2.pdf]

Supplementary Figure 2. Barplots for the normal vs. AMI and AMI post comparison. The proteins shown in this figure are the 35 proteins that were significantly different (log2 FC>1; q<0.05) in the normal vs. AMI comparison. Normal (n=6), AMI (n=12), AMI post (n=4).

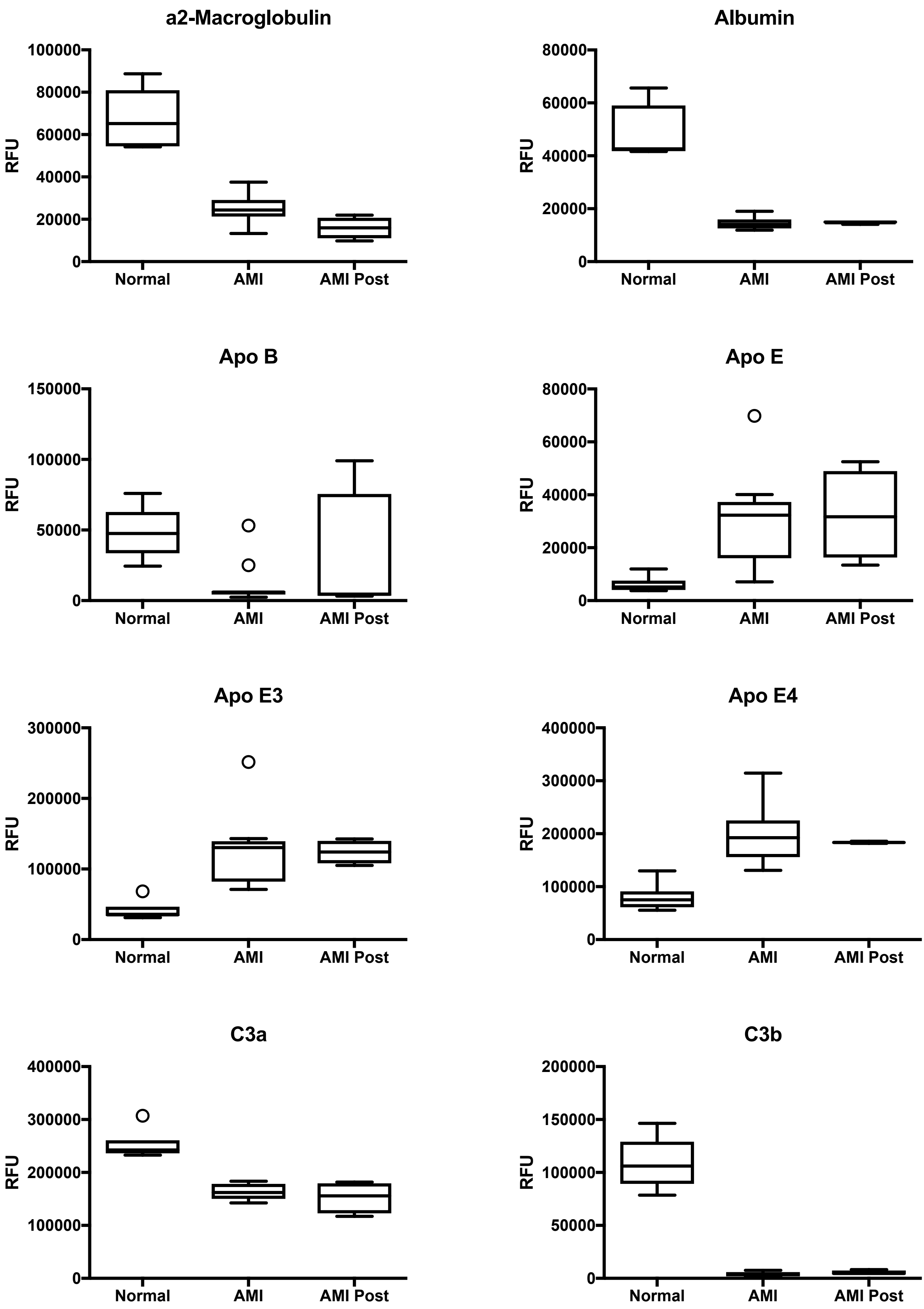
a2-Macroglobulin
Albumin
Apo B
Apo E
Apo E3
Apo E4
C3a
C3b
RFU
Normal
AMI
AMI Post

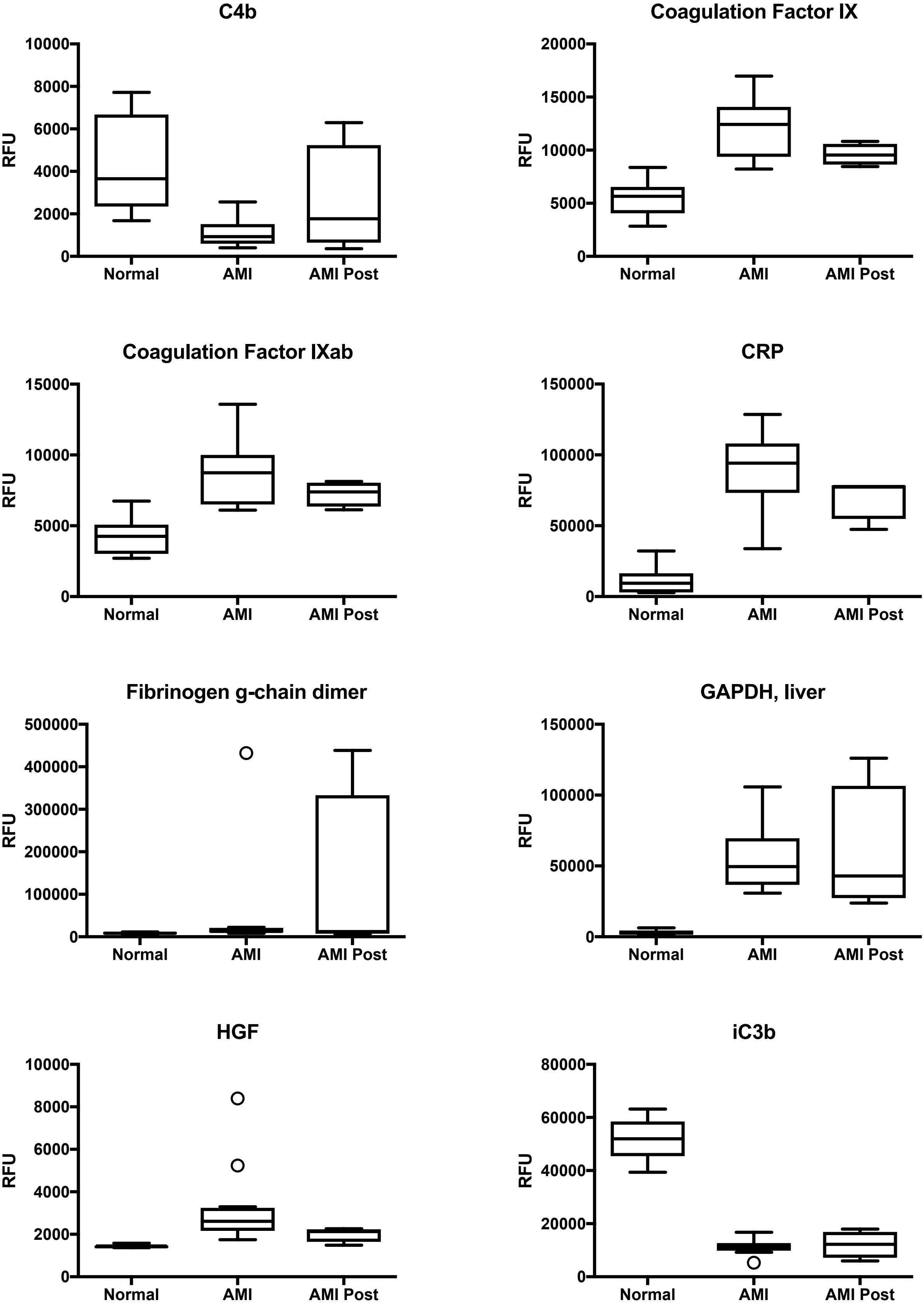
C4b
Coagulation Factor IX
Coagulation Factor IXab
CRP
Fibrinogen g-chain dimer
GAPDH, liver
HGF
iC3b
RFU
Normal
AMI
AMI Post
0
2000
4000
6000
8000
10000
5000
15000
20000
50000
100000
150000
200000
300000
400000
500000
20000
40000
60000
80000

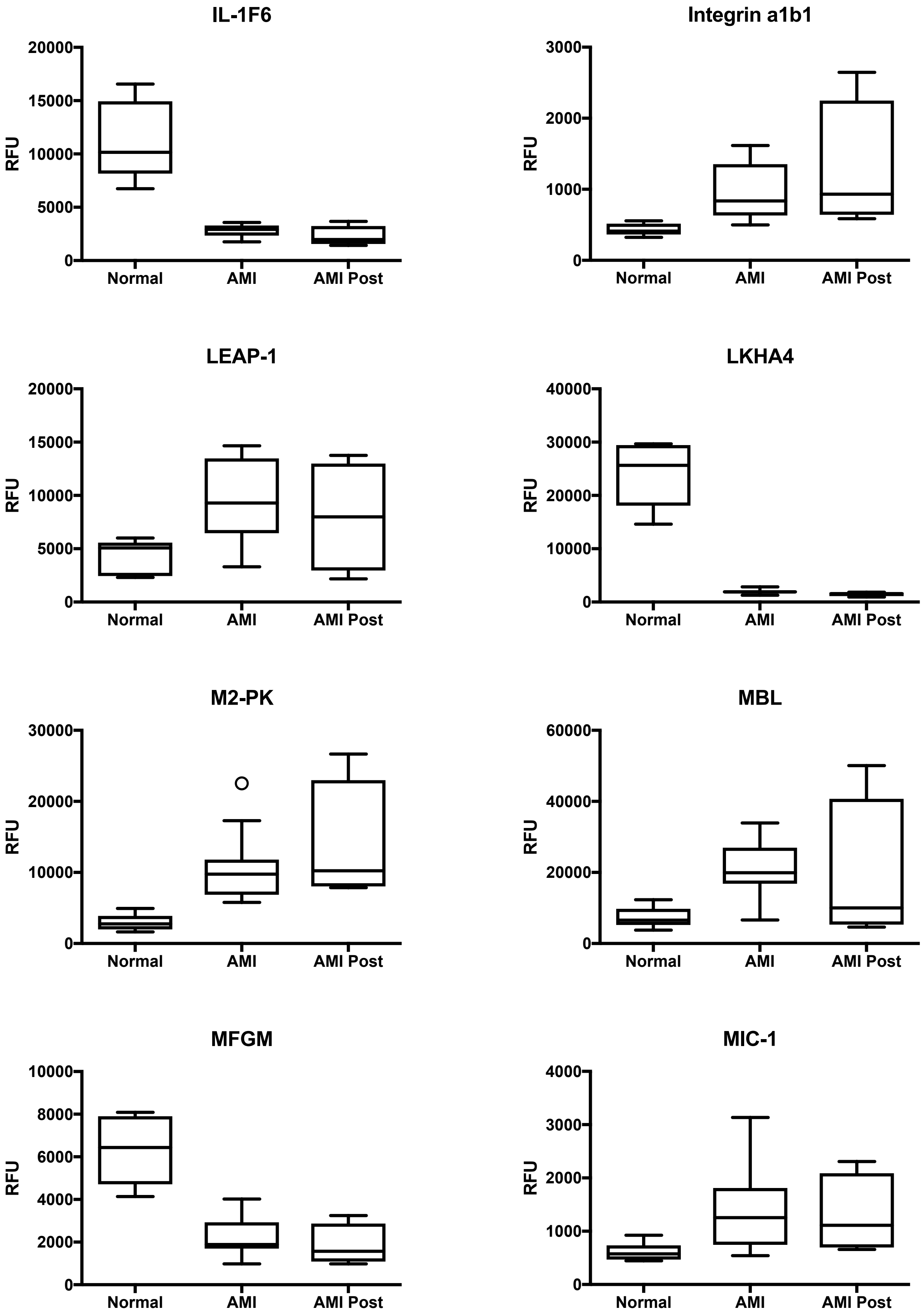
IL-1F6
RFU
20000
15000
10000
5000
0
Normal
AMI
AMI Post
Integrin a1b1
RFU
3000
2000
1000
0
Normal
AMI
AMI Post
LEAP-1
RFU
20000
15000
10000
5000
0
Normal
AMI
AMI Post
LKHA4
RFU
40000
30000
20000
10000
0
Normal
AMI
AMI Post
M2-PK
RFU
30000
20000
10000
0
Normal
AMI
AMI Post
MBL
RFU
60000
40000
20000
0
Normal
AMI
AMI Post
MFGM
RFU
10000
8000
6000
4000
2000
0
Normal
AMI
AMI Post
MIC-1
RFU
4000
3000
2000
1000
0
Normal
AMI
AMI Post

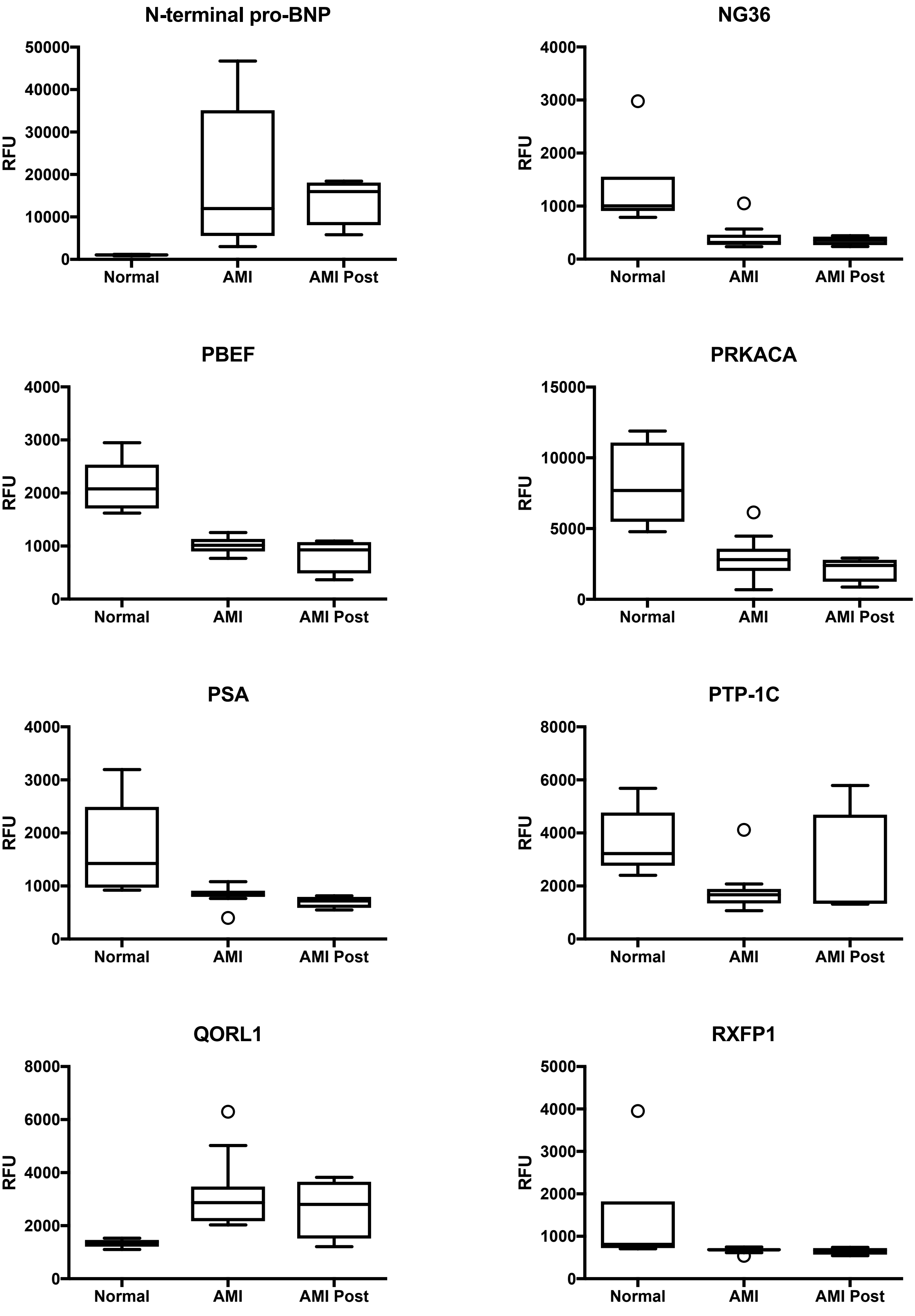
N-terminal pro-BNP
RFU
50000
40000
30000
20000
10000
0
Normal
AMI
AMI Post
NG36
RFU
4000
3000
2000
1000
0
Normal
AMI
AMI Post
PBEF
RFU
4000
3000
2000
1000
0
Normal
AMI
AMI Post
PRKACA
RFU
15000
10000
5000
0
Normal
AMI
AMI Post
PSA
RFU
4000
3000
2000
1000
0
Normal
AMI
AMI Post
PTP-1C
RFU
8000
6000
4000
2000
0
Normal
AMI
AMI Post
QORL1
RFU
8000
6000
4000
2000
0
Normal
AMI
AMI Post
RXFP1
RFU
5000
4000
3000
2000
1000
0
Normal
AMI
AMI Post

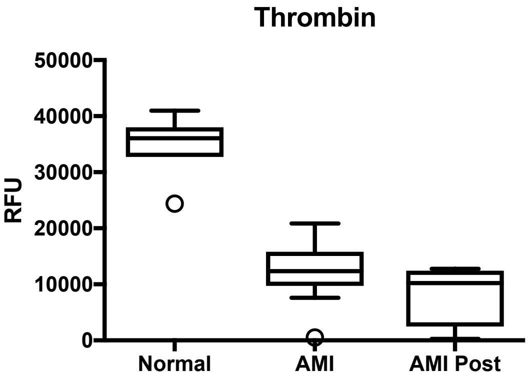
Thrombin
50000
40000
30000
20000
10000
0
RFU
Normal
AMI
AMI Post

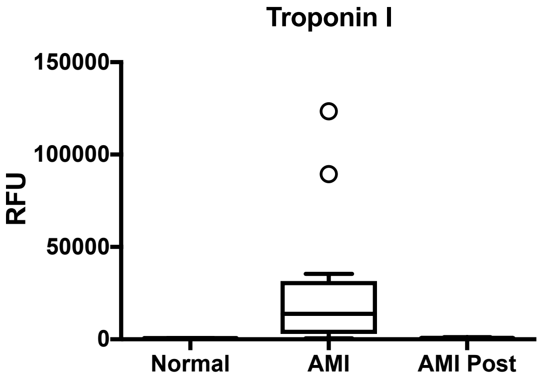
Troponin I
150000
100000
50000
0
RFU
Normal
AMI
AMI Post

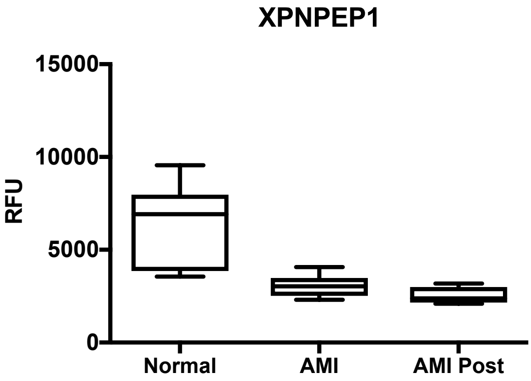
XPNPEP1
15000
10000
5000
0
RFU
Normal
AMI
AMI Post
